# Image-based SPECT calibration based on the evaluation of the Fraction of Activity in the Field of View

**DOI:** 10.1186/s40658-018-0209-8

**Published:** 2018-05-16

**Authors:** Adrien Halty, Jean-Noël Badel, Olga Kochebina, David Sarrut

**Affiliations:** 1 Univ Lyon, INSA-Lyon, Université Lyon 1, CNRS, Inserm, CREATIS UMR 5220, U1206, Lyon, 69008 France; 2 Univ Lyon, Centre Léon Bérard, Lyon, 69008 France

**Keywords:** Targeted radionuclide therapy, Absorbed dose estimation, SPECT calibration

## Abstract

**Background:**

SPECT quantification is important for dosimetry in targeted radionuclide therapy (TRT) and the calibration of SPECT images is a crucial stage for image quantification. The current standardized calibration protocol (MIRD 23) uses phantom acquisitions to derive a global calibration factor in specific conditions. It thus requires specific acquisitions for every clinical protocols. We proposed an alternative and complementary image-based calibration method that allows to determine a calibration factor adapted to each patient, radionuclide, and acquisition protocol and that may also be used as an additional independent calibration.

**Results:**

The proposed method relies on a SPECT/CT acquisition of a given region of interest and an initial whole-body (WB) planar image. First, the conjugate view of WB planar images is computed after scatter and attenuation correction. 3D SPECT images are reconstructed with scatter, attenuation, and collimator-detector response (CDR) corrections and corrected from apparent dead-time. The field of view (FOV) of the SPECT image is then projected on the corrected WB planar image. The fraction of activity located in the area corresponding to the SPECT FOV is then calculated based on the counts on the corrected WB planar image. The Fraction of Activity in Field Of View (FAF) is then proposed to compute the calibration factor as the total number of counts in the SPECT image divided by this activity. Quantification accuracy was compared with the standard calibration method both with phantom experiments and on patient data.

Both standard and image-based calibrations give good accuracy on large region of interest on phantom experiments (less than 7% of relative difference compared to ground truth). Apparent dead-time correction allows to reduce the uncertainty associated with standard calibration from 2.5 to 1%. The differences found between both methods were lower than the uncertainty range of the standard calibration (<3*%*). In patient data, although no ground truth was available, both methods give similar calibration factor (average difference 3.64%).

**Conclusions:**

A calibration factor may be computed directly from the acquired SPECT image providing that a WB planar image is also available and if both acquisitions are performed before biological elimination. This method does not require to perform phantom acquisition for every different acquisition conditions and may serve to double check the calibration with an independent factor.

## Background

In targeted radionuclide therapy (TRT), the determination of the spatial and the temporal radioactivity distributions within the body is required to estimate the absorbed dose distribution. In practice, in vivo activity distributions can be visualized from 3D SPECT images. Currently, SPECT has become an imaging modality as quantitative as PET [1], although SPECT images are generally noisier. Indeed, images are degraded by several phenomena: photons attenuation and scattering, instrumentation constraints such as partial volume effects (PVE) or dead-time (DT), and motion artifact [2]. A lot of efforts have been conducted toward a reliable quantification [2–6]. Nowadays, scatter and attenuation corrections are embedded into most reconstruction algorithms, PVE correction is partly tackled with recovery coefficient, and DT correction is feasible. For example, a global 5% accuracy of ^99m^Tc quantification has recently been reported which is similar to one obtained with ^18^F in PET [7]. On in vivo data, a standard error of 8.4% has been reported in bladder activity quantification [6]. Similar results (standard error of 7%) were found on corrected ventilation-substracted perfusion images [5].

However, even if significant progresses have been made during the last 10 years [8], reliable quantification remains difficult. In particular, a crucial step is the determination of a global calibration factor of the system sensitivity that convert a number of counts (cts) into an activity concentration in Bq (per voxel). The MIRD committee recommends [4] to perform the acquisition of a known activity with scattering condition close to presented with a patient. The exact same parameters of acquisition (energy windows) and reconstruction must be used in patient data. This means that a calibration factor should be determined each time when one of the parameters, such as the width or the number of energy windows for multi-gamma emitters or the acquisition duration, is changed. The acquisition parameters depend on specific clinical needs and constraints of a study and thus may require a dedicated calibration factor. This is sometimes inconvenient in clinical routine and could be costly for expensive radionuclides such as ^177^Lu or ^111^In.

In this work, we propose an image-based method, similar to one sometimes used in radioembolization dosimetry [9–11], where the total injected activity is present in the FOV of the SPECT. This method is often preferred to the MIRD method because it is patient-specific. The proposed approach is a generalization for the cases where the injected activity is not entirely present in the SPECT FOV. It consists in an estimation of the calibration factor from the image itself, using the apparent fraction of activity in the field of view and the known injected activity. In the following, we first describe the proposed method and then provide the experimental results of experiments in order to evaluate its performances.

## Methods

SPECT voxel values are typically expressed in number of counts (cts), and the quantification goal is to determine the calibration factor *S* (or system sensitivity) to convert cts to Bq. It is important to keep in mind that the calibration factor is global and it does not take into account PVE effects, and coefficient recovery factors are still required to perform the quantification on small volumes.

The current MIRD guidelines are briefly reviewed before describing the proposed image-based method.

### Phantom-based calibration according to MIRD guidelines

According to the MIRD guidelines for the quantification [4, 12], the calibration factor *S*_std_ (std for “standard”) is computed using a SPECT acquisition of a large source of a known activity in a determined phantom. Typically a large uniform tank (Jaszczak phantom) of water with a low activity concentration (similar to what is expected in clinic) is imaged with the exact same parameters as in the clinical study where the calibration factor will be used. SPECT images should be reconstructed with a method that includes attenuation correction (AC) based on CT [13], scatter correction (SC) based on double- or triple-energy window (DEW or TEW) methods [14], and collimator-detector response (CDR) compensation [15]. Although less frequently mentioned in publications, images should be corrected from *apparent* DT (aDT) [16] and not only the *system* DT (sDT). Indeed, *system* DT, noted *τ* is often relatively low in the modern devices (*τ*≈ 1 or 2 *μ*s [3, 5]). Willowson et al. [5] reported that a significant impact of sDT may be observed for count rates of 40 kcps or higher, corresponding to a theoretical loss of 5%. However, aDT can be significantly higher than sDT; indeed, all detected events regardless of their energy cause DT and not only counts recorded in the primary or scatter windows [12]. The aDT, denoted *τ*_a_, is then given by Eq. 1 where the window fraction *ω*_f_ corresponds to the percentage of detected events in the energy windows of interest (photopeaks, scatter).

1 $$ \tau_{\mathrm{a}}=\frac{\tau}{\omega_{\mathrm{f}}}  $$

We denoted *A*_mean_ the mean activity over the acquisition duration *ΔT*_acq_ given by Eq. 2.

2 $$ {A}_{\text{mean}} =\frac{A_{0}\int_{0}^{\Delta T_{\text{acq}}} e^{-\lambda t} \, \mathrm{d}t }{\Delta T_{\text{acq}}}  $$

Here, *A*_0_ is the injected activity corrected from the potential residual activity in syringes and physical decay.

Therefore, the calibration factor *S*_std_ is given by Eq. 3, with *N*_SPECT_ the total number of counts in the SPECT image corrected by SC, AC, CDR, and DT.

3 $$ S_{\text{std}}=\frac{N_{\text{SPECT}}}{A_{\text{mean}} \times \Delta T_{\text{acq}}}  $$

The repeatability of the standard calibration factor, *S*_std_, was estimated from several measurements with the coefficient of variation, COV, given by Eq. 4, $\overline {S_{\text {std}}}$ being the average *S*_std_ over the experiments.

4 $$ \text{COV}=\frac{\sigma_{S_{\text{std}}}}{\overline{S_{\text{std}}}}  $$

The MIRD methodology hence requires rigorous experiments and specific logistics (cost and storage of phantoms with long half-live radionuclides). Consequently, it is not always easy to implement in clinical routine despite the good results obtained in terms of accuracy.

### Calibration factor with all activity inside the field of view

A specific situation may be considered when the total injected activity is inside the SPECT field of view (FOV). In this case, the standard phantom acquisition is not needed anymore. The image-based calibration factor *S*_imb_ is directly derived from the patient image with Eq. 5, in the assumption that the total injected activity is inside the patient. This approach is often used in radioembolization dosimetry [9–11]. For example, Paciolia et al. have recently reported that relative calibration allows to partially compensate suboptimal scatter corrections.

5 $$ S_{\text{imb}}=\frac{N_{\text{SPECT}}}{A_{mean} \times \Delta T_{\text{acq}} }  $$

### Calibration factor with activity inside and outside the field of view

Most of the times, the injected activity is spread in the blood flow and therefore is not entirely present in the SPECT FOV that is generally centered in the region of interest. This is, for example, the case for pre-therapeutic dosimetry with ^177^Lu treatment of neuroendocrine tumors [17], administrated by intravenous injections. In this case, the previously described assumption is not fulfilled, and Eq. 5 cannot be applied. Moreover, performing SPECT acquisitions with several table steps to cover the whole body would be generally too long in the clinical routine. Instead, we propose to estimate the activity in the SPECT FOV thanks to the planar whole-body scintigraphies (WBS). WBS acquisitions are generally significantly faster than tomographic SPECT acquisition (about 10 cm/min for WBS versus the equivalent of 2.5 cm/min for SPECT). WBS are generally acquired just before SPECT to adjust the table position. The proposed method consists in the following steps:

1. Acquisition of conjugate planar WBS with scatter and attenuation correction.
2. Projection of the SPECT FOV boundaries on WBS.
3. Computation of the Fraction of Activity in FOV (FAF) and of the calibration factor S

**Step 1** Conjugate planar WBS images are first corrected for scatter with the DEW method [14]. Anterior and posterior images are then combined with the geometric mean method [18]. The CT image of the SPECT/CT acquisition is converted into a 3D attenuation coefficient map, which is then averaged along the antero-posterior (AP) axis to obtain a 2D mean attenuation map with the same spatial orientation as the scatter corrected planar images. Finally, the 2D planar image is corrected for attenuation. If the CT image is smaller than conjugate planar WBS, the attenuation coefficient factor is extrapolated from the border of the planar attenuation coefficient map.

**Fig. 1:**
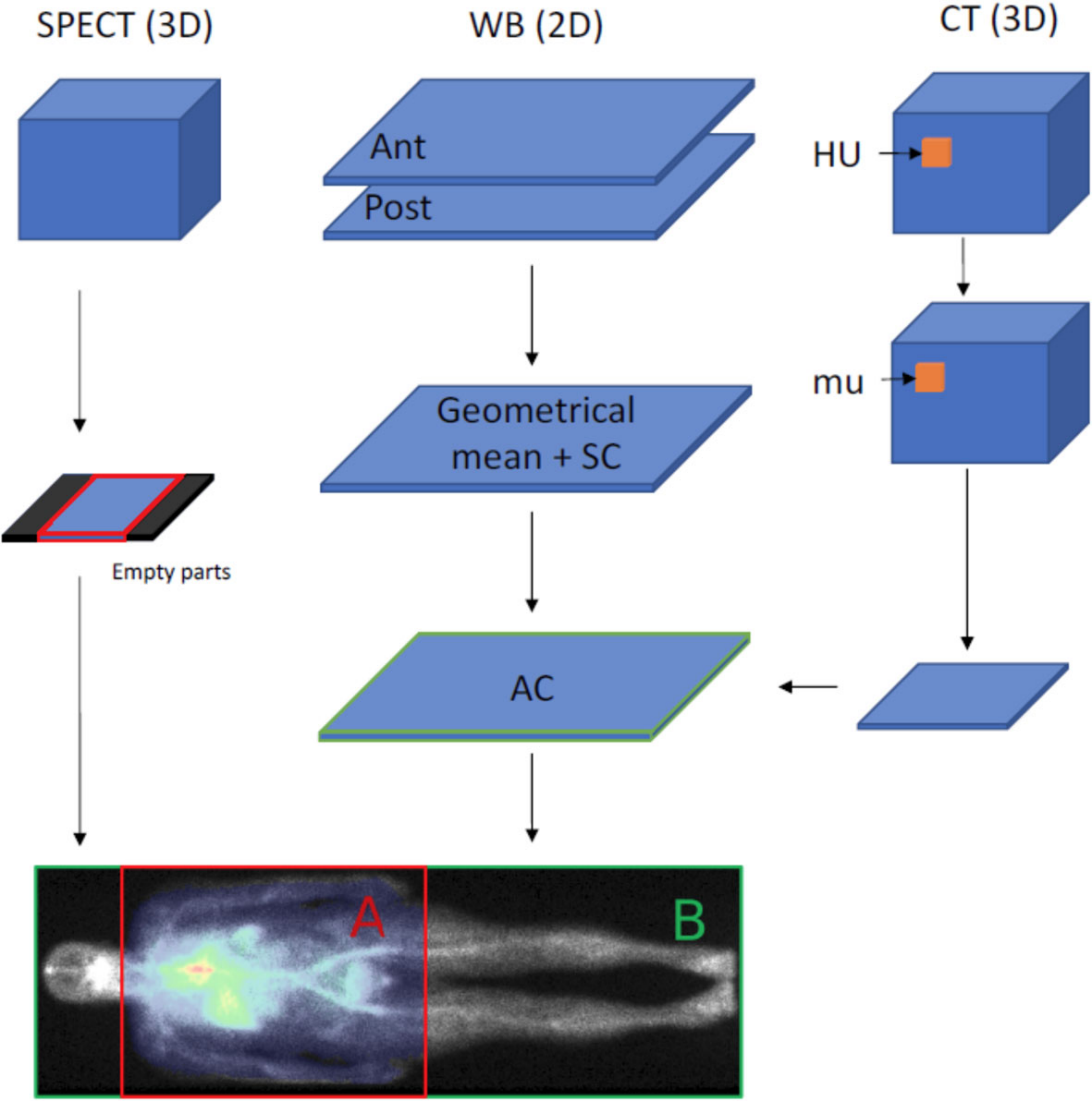
SPECT image is projected along the antero-posterior axis

**Step 2** The boundaries of the SPECT FOV are projected along the AP axis onto the 2D planar image as shown in Fig. 1. This step may require to align the 3D SPECT and the 2D planar images if they are in different coordinate systems. This registration could be performed using table coordinates in the DICOM files or by automated rigid 2D registration between planar image and AP-projected SPECT image. On some devices, the SPECT voxel matrix size may be larger than the real detector length leading to two bands of voxels with zero counts in the top and bottom parts of the image. Those bands must be removed before projecting the boundaries onto the planar image, see Fig. 1.

**Step 3** The ratio between the number of counts *N*_A_ within the 2D ROI (region named “A” in Fig. 1) obtained after Step 2, and the total number of counts *N*_B_ in the whole WBS (region named “B” in the figure) is defined as the Fraction of Activity in FOV (FAF) factor, $\text {FAF} = \frac {N_{\mathrm {A}}}{N_{\mathrm {B}}}$. The final calibration factor is then obtained with Eq. 6 where *N*_SPECT_ is the number of counts in the 3D SPECT image and *A*_mean_ is the averaged injected activity over the acquisition (2).

6 $$ S_{\text{FAF}}=\frac{N_{\text{SPECT}}}{A_{\text{mean}} \times \Delta T_{\text{acq}} \times \text{FAF} }  $$

This method requires the totality of injected activity being in the patient body. Therefore, the images (planar WB and SPECT/CT) must be acquired before any biological elimination (e.g., before urination). This approach is based on the assumption that the fraction of activity present in the 3D SPECT FOV can be estimated from the 2D planar images.

## Experiments

Several experiments were performed to evaluate the proposed methods. All experiments used ^99m^Tc, but the method could be applied to other radionuclides. An example of the use of this method for a TRT with ^111^In is given in the last part.

### Imaging acquisition and reconstruction for the ^99m^Tc experiments

The image acquisitions were performed on a Tandem Discovery NM/CT 670 from GE Medical Systems with two heads. We used LEHR/PARA collimators (low-energy high-resolution/parallel) with hexagonal holes. The head radius was set to a constant distance of 24 cm. Two energy windows, primary and scatter, were recorded with the standard clinical settings. The primary window corresponding to the photopeak of ^99m^Tc and was set to 126.45–154.55 keV, and the scatter window was set to 114–126 keV. SPECT acquisitions consisted in 60 step-and-shoot projections of 25 s each and over 360°. The spatial sampling was 4.418 × 4.418 mm, and the 2D matrix of pixel was 128 × 128. CT was acquired right after SPECT, with a tube voltage of 120 kV. Slice thickness was 1.25 mm, and pixel spacing was 0.9765 × 0.9765 mm. SPECT reconstruction was performed with manufacturer’s iterative ordered-subset expectation maximization (OSEM) algorithm that include attenuation, DEW scatter, and CDR correction. All images were reconstructed with the same software version (Xeleris 3.0) and parameters sets, with 10 subsets and 20 iterations.

### Standard phantom-based MIRD calibration

First, the standard calibration factor, *S*_std_, was computed from the phantom-based MIRD calibration procedure from repeated SPECT/CT acquisitions of a Jaszczak phantom with three spheres of high concentration of ^99m^Tc (10 MBq in total), respectively of 16, 8, and 4 mL. The spheres were placed in uniform background with several increasing activity concentrations. The background activity concentrations were 10, 20, 30, and 50% of the sphere activity concentration. Values are reported in Table 1 after correction from residual activity in syringes and physical decay. The different levels of activity allowed to evaluate the DT correction. Four acquisitions were performed at each level of background activity to estimate the reproducibility.

**Table 1:** Activities for standard calibration phantom acquisitions

Acquisition	Spheres	Phantom	Background to sphere
number	activity (MBq)	activity (MBq)	concentration ratio (%)
1–4	9.46	247.47	10.20
5–8	9.46	491.47	20.67
9–12	9.46	743.49	31.47
13–16	9.46	1238.38	52.69

The value of *S*_std_ was estimated according to the MIRD guidelines. Large ROI covering about 2 cm around the phantom were manually drawn to compensate from spill-out effects.

### Dead-time correction

A paralyzable model was considered; a new event resets the time frame of no detection. This model is described by Eq. 7 with *τ* the sDT, *R*_o_ the observed count rate, and *R*_t_ the true count rate without dead-time [16].

7 $$ R_{\mathrm{o}}=R_{\mathrm{t}}e^{-R_{\mathrm{t}} \tau}  $$

Dead-time of camera was experimentally measured with the two-sources method [16]. Three acquisitions were performed with an energy window set to 0–511 keV in order to record the whole energy spectrum. The first acquisition was performed without collimator, with a 40-MBq source. In the second acquisition, another source of 40-MBq was added next to the first one. The third acquisition was done keeping only the second one. The count rate was evaluated for each acquisition and denoted respectively *R*_1_, *R*_12_, and *R*_2_. The dead-time, *τ*, is calculated through them as shown in Eq. 8 [16].

8 $$ \tau\approx\frac{2R_{12}}{(R_{1}+R_{2})^{2}}\text{ln}\left(\frac{R_{1}+R_{2}}{R_{12}}\right)  $$

The phantom used for the standard phantom-based calibration was placed in the same position as in the calibration experiments and with an additional window set to 0–511 keV. The window fraction, *ω*_f_, was computed as the ratio between the primary and scatter counts and the counts recorded in the total spectrum window. The value of aDT was computed with Eq. 1. Equation 7 was solved with *τ* for sDT correction, or *τ*_a_ for aDT correction. Reconstructed images were then scaled by $\frac {R_{\mathrm {t}}}{R_{\mathrm {o}}}=e^{R_{\mathrm {t}} \tau } \left (\text {or} = e^{R_{\mathrm {t}} \tau _{\mathrm {a}}}\right)$.

### Test case 1: Accuracy of image-based method

**Fig. 2:**
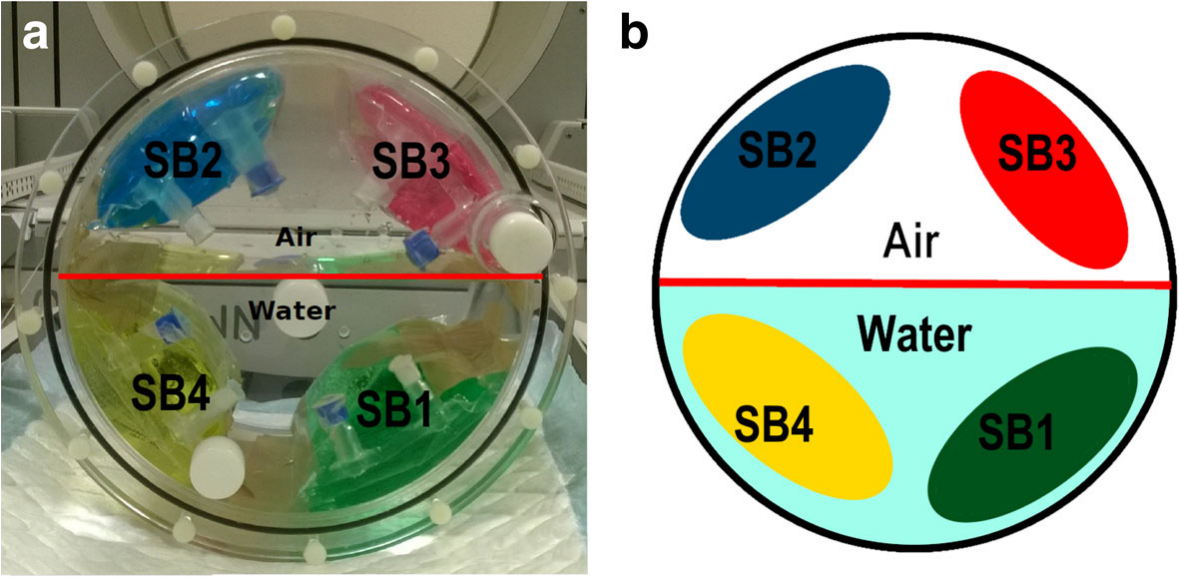
Photo (**a**) and schematic view (**b**) of the phantom used to test calibration with different conditions of attenuation and scattering. Here, two saline bags are in attenuating condition (water) and two in non-attenuating condition (air) corresponding to the third acquisition

Test case 1 was designed to compare *S*_std_ obtained from conventional phantom-based calibration with *S*_imb_ obtained from our image-based method, when all activity is in the FOV. Four bags of 500 mL of saline solution containing ^99m^Tc were placed in a cylindrical phantom half filled with water without activity, see Fig. 2. It allows to evaluate the different conditions of attenuation and scatter as some bags were in the water while others were in the air. In the acquisitions 1 and 2, two bags were placed at the air/water interface. Hence, about half of the saline bag was in the air and the other half was in the water. The activities in the saline bags are given in Table 2. Residual activities in the syringes after injection were taken into account.

**Table 2:** Activities for image-based quantification test

Saline bag	Color	Activity (MBq)	Position in acquisition 1	Position in acquisition 2	Position in
					acquisition 3
1	Green	54.52	Interface	Air	Water
2	Blue	59.32	Interface	Water	Air
3	Red	111.33	Air	Interface	Air
4	Yellow	111.99	Water	Interface	Water

The acquisition protocol and reconstruction parameters were identical to those used in the standard phantom-based MIRD calibration method. A calibration factor, named *S*_imb_, was determined from the image, knowing that all injected activity was visible in the SPECT FOV.

The bag ROIs were manually selected on the images as spheres of 3 cm of diameter inside the bag contour, away from the boundaries of the bag in order to avoid PVE. The activities in the four bags were determined as the mean activity in bag ROIs based on the two CFs and compared to the known ground truth values. The relative error on quantification was calculated as the difference between the activity found with the considered calibration factor and the ground truth, divided by the ground truth value.

### Test case 2: FAF evaluation

**Fig. 3:**
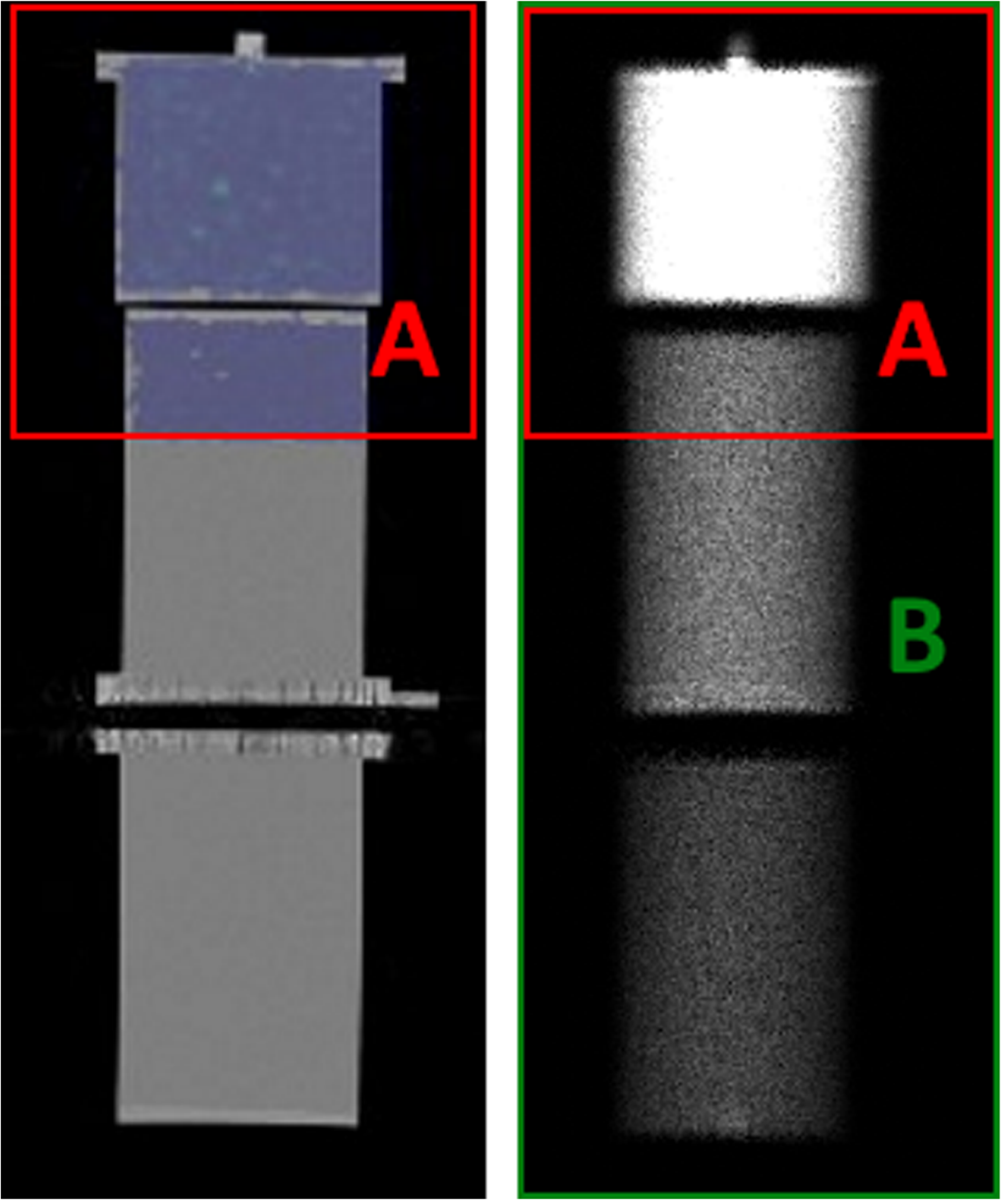
Projection of 3D SPECT into the 2D WBS on phantom. The area A in red is obtained from the antero-posterior projection of the SPECT FOV into the whole-body planar image. The area B corresponds to the entire WB planar image

Test case 2 was designed to evaluate the hypothesis used for the FAF method. Image acquisitions were performed on a phantom composed of three parts. The first one consists of three spheres of respectively 4, 8, and 16 mL with a high activity concentration of ^99m^Tc placed inside a Jaszczak phantom with medium activity concentration. The two other parts are cylindrical phantoms filled with water of low activity concentration placed next to the Jaszczak phantom. The spheres in the Jaszczak phantom mimic thoracic lesions, while the cylindrical phantoms mimic the lower limbs. SPECT acquisitions were performed with a FOV that cover entirely the Jaszczak phantoms and partly one cylindrical phantom as shown in Fig. 3. This experiment was performed with three activity concentrations, denoted 2a, 2b, and 2c as summarized in Table 3. The acquisition parameters and reconstruction protocols were the same than detailed for the standard calibration. For the WBS acquisition, the spatial sampling was 2.209 × 2.209 mm, and the 2D matrix of pixel was 256 × 1024. The total acquisition time was 4 min.

**Table 3:** Activities used for test case 2

Experiment	Sphere	Jaszczak	Cylinder 1	Cylinder 2	Fraction of
	concentration	concentration	concentration	concentration	cylinder 1 in
	(kBq/mL)	(kBq/mL)	(kBq/mL)	(kBq/mL)	SPECT FOV(%)
2a	158.9	15.58	2.76	3.06	50
2b	140.85	14.12	4.68	2.56	32
2c	126.95	12.73	2.30	4.21	23

The 2D planar image scatter correction was performed with the DEW method [14] as recommended by the manufacturer; a scatter multiplier of 1.1 was used to scale the number of counts in the scatter window according to the number of scattered photons in the photopeak window. The attenuation correction was also performed according to the manufacturer’s formula, reported, and validated in [13], see Eq. 9, with *μ*_*m*_, *μ*_*w*_, *μ*_*a*_, and *μ*_*b*_ the attenuation coefficients of respectively material, water, air, and bone, *E* the energy of the gamma photons in keV, and *E*_eff_ the mean energy of the CT beam. We assume $E_{\text {eff}}= \frac {E_{\text {peak}}}{3}$, with *E*_peak_ the maximum energy of the CT beam.

9 $$\begin{array}{*{20}l} \mu_{m, E}=\mu_{w, E} + \frac{\left(\mu_{w, E}-\mu_{a, E}\right)\times \text{CT}}{1000} \qquad \text{if CT} < 0\\ \mu_{m, E}=\mu_{w, E} + \frac{\mu_{w, E_{\text{eff}}}\times\left(\mu_{b, E}-\mu_{w, E}\right)\times \text{CT}}{1000\times\left(\mu_{b, E_{\text{eff}}}-\mu_{w, E_{\text{eff}}}\right)} \qquad \text{if CT} > 0 \end{array} $$

### Test case 3: Patient study

The proposed image-based calibration method was applied to clinical patient images. We compared *S*_FAF_ to *S*_std_ obtained from a uniform Jaszczak phantom acquisition with an activity of 13.64 MBq of ^111^In at the time of acquisition, according to the MIRD protocol.

We selected images of six patients from a phase I clinical trial named Synfrizz which was previously approved by local authorities (ANSM; ClinicalTrials.gov Identifier: NCT01469975). It involved a ^90^Y radiolabeled monoclonal antibody (mAb), OTSA101, developed by OncoTherapy Science (OTS) targeting a tumor antigen over-expressed in synovial sarcoma [19]. Before the therapy, patients were injected with ^111^In-labeled mAb to evaluate uptakes and biodistributions. Sequences of planar WBS, immediately followed by SPECT/CT images, were acquired at 1, 5, 24, 48, 72, and 144 h following the intravenous injection.

**Fig. 4:**
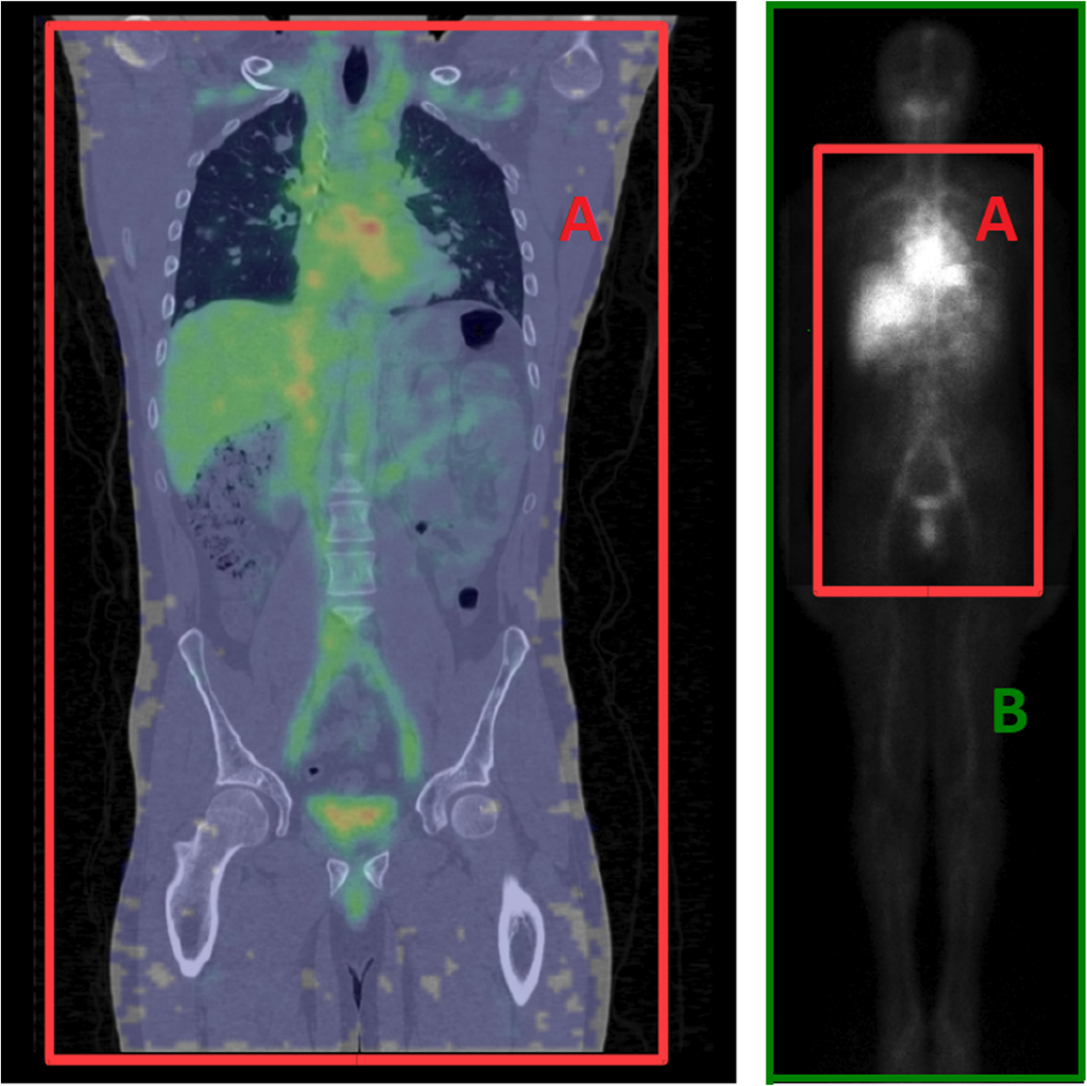
On the left side, SPECT/CT fusion image of a Synfrizz patient. On the right, whole body planar image after attenuation and scatter correction. The SPECT FOV is represented on both image by the red rectangle

The imaging protocol was similar to the previous test cases, except that the MEGP/PARA (medium-energy general-purpose/parallel) collimators with hexagonal holes were used. ^111^In has two main gamma ray emissions at 171 and 245 keV. As recommended by the manufacturer, the primary energy windows were 153.9–188.1 keV and 220.5–269.5 keV, and the scatter window used for DEW scatter correction was 198.3–219.6 keV. SPECT acquisitions consisted in 60 step-and-shoot projections of 30 s each and over 360° followed by CT aquisition. SPECT images were reconstructed with the manufacturer OSEM algorithm provided by the software (Xeleris 3.0). In addition to scatter correction with DEW, attenuation correction based on CT image and “resolution recovery” package were used. SPECT voxel spacing was 4.18 × 4.18 × 4.18 mm^3^. For the dead-time correction, similar dead-time as in phantom study was assumed. Indeed, the whole spectrum was not recorded at the time of data acquisition to allow proper correction of aDT. The SPECT acquisition was performed with two table steps, with a small overlap, covering in total 92 cm from the patient’s neck to below the pelvic region, see Fig. 4. Here, *S*_FAF_ was applied on a two-step image, and *A*_mean_ is the mean activity during one step. Because of physical decay, the *A*_mean_ of the second step is slightly lower. Therefore, the average *A*_mean_ between the two steps was used to compute *S*_FAF_.

The WBS planar image dimension was 1024 × 256 with pixel spacing of 2.40 × 2.40 mm^2^. Table velocity was 10 cm/min. *S*_FAF_ was estimated with the method described above on the first acquired SPECT image, 1 h after injection. No biological elimination occurred between injection and the first image acquisition. Time between WBS and SPECT/CT was always less than 10 min. The total activity *A*_mean_ in patients was equal to the injected activity corrected from decay and residual activity of the syringe as given by Eq. 2. In the clinical study, the heart, the kidneys, the liver, the spleen, the bone marrow, and the main lesions were analyzed [19]. However, since the activity ground truth in each ROI is unknown, only the difference in the global calibration factor was considered.

## Results

### Standard calibration factor with ^99m^Tc

Measured sDT of the GE camera was 1.66 *μs*, and the window fraction on this configuration was 46% resulting in an aDT of 3.6 *μs*. With sDT, correction factors were from 1.015 on the last 10% background acquisition (with the lowest count rate) to 1.048 on the first 50% background acquisition (with the highest count rate). With aDT, correction factor ranges were larger than those with sDT. They were from 1.034 to 1.112 in the same conditions. In other words, in the highest count rate configuration, we assume that 11.2% of events are lost with aDT, compared to only 4.8% with sDT.

**Fig. 5:**
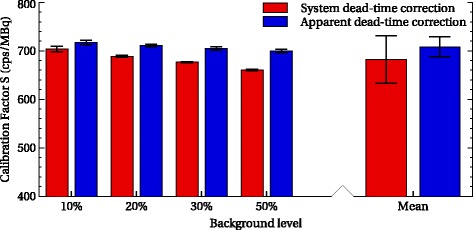
Calibration factor and uncertainties with correction of aDT (blue) and and sDT (red). Error bars correspond to 3*σ* on repeated measurements

Whatever the level of background, the *S*_std_ values remained stable with a coefficient of variation in the range 0.11–0.23%, showing a good repeatability. The calibration factor was found to be 708 cps/MBq with a COV of 0.96% with aDT correction. Note that the value was 682 cps/MBq with a COV of 2.39% with sDT correction. Figure 5 displays the *S*_std_ values for the different configurations and the associated uncertainty (3*σ* of repeated measurements). We observed a slight decrease of 2.54% of the calibration factor when the background level increases, i.e., with the increasing count rate. In the following, only aDT calibration is considered and compared to the image-based method.

### Test case 1: Evaluation of image-based method on phantoms experiments

**Fig. 6:**
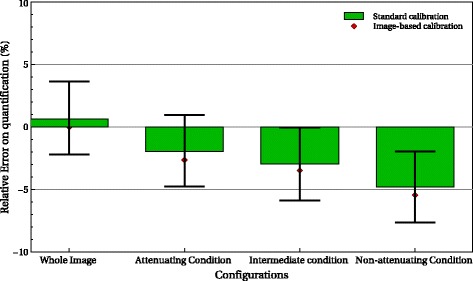
Quantification error in the different configurations and regions with the image-based method (red) and the standard calibration (green)

As expected when all the activity is inside the FOV, results with the image-based method are similar to those with the standard calibration. Figure 6 gives the error on quantification with both methods, on the whole image and different bags. We grouped the bags according to the attenuation condition: attenuating, non-attenuating, and intermediate. On the whole image, the image-based method leads to no quantification error by construction. We considered all the activities in the FOV to calculate *S*_imb_. With the standard method, the relative error is also very small (0.65%). In the subregions of interest, both methods give relative errors less than 6%. The difference of relative errors between the two methods was small (0.54–0.68%) compared to the uncertainty associated to the standard method (± 3*%*).

### Test case 2: Evaluation of the FAF method

**Fig. 7:**
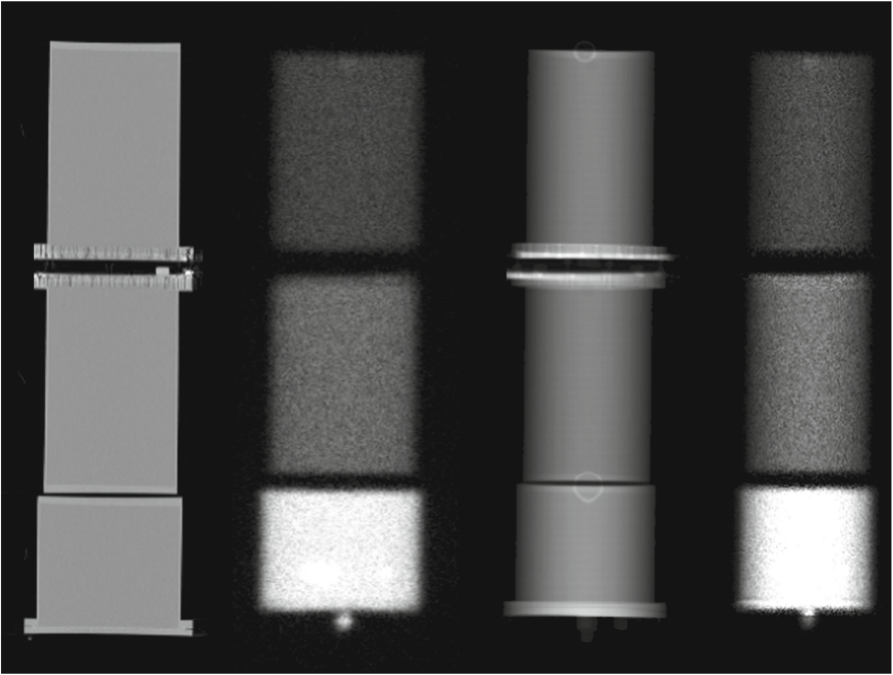
Illustration of the AC in the WBS. From left to right, a coronal view of the phantoms used, the geometrical mean of the planar scintigraphy corrected from SC, the attenuation correction factor map obained from the CT, and the WBS corrected from scatter and attenuation

**Fig. 8:**
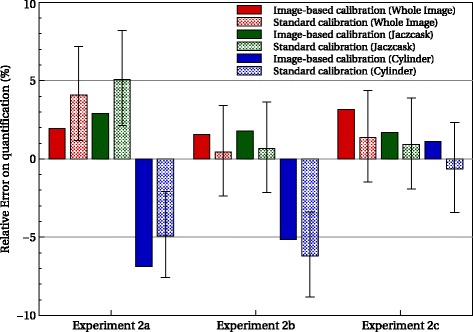
Quantification error for the different configurations and ROI with the image-based FAF method (filled) and the standard calibration (dot) compared to the ground truth

Figure 7 illustrates the attenuation correction of the WBS. Table 4 shows the estimated and real FAF for the three experiments 2a, 2b, and 2c. Absolute errors were less than 2% in all configurations. Figure 8 depicts the differences between standard- and image-based FAF quantification for the three experiments used for three ROIs: the whole image, the Jaszczak phantom part, and the cylinder. The relative errors compared to ground truth range from -6.18 to 5.08% with the standard calibration method and from - 6.87 to 3.16% with image-based FAF method. Again, the differences between both methods are within the uncertainty of standard calibration (± 3*%*).

**Table 4:** Evaluation of fraction of activity in the FOV test

Experiment	True FAF (%)	Estimated FAF (%)	Absolute error (%)
2a	74.93	76.39	1.46
2b	68.04	69.10	1.06
2c	63.07	65.06	1.99

### Test case 3: Patient study

The calibration factor obtained with the standard method was 1352 cps/MBq. The image-based calibration factors as well as the relative difference with the standard method are given in Table 5. The average difference was 3.64% with a standard deviation of 4.46%. Differences up to 9% were observed.

**Table 5:** Comparison of the calibration factors obtained with standard method and image-based FAF method, computed on patient data

Patient	*S*_FAF_ (cps/MBq)	Rel. diff. with *S*_std_(%)	Arms in same position
1	1335	-1.29	Yes
2	1411	4.19	No
3	1374	1.58	No
4	1481	8.72	No
5	1347	-0.39	Yes
6	1486	9.00	No

No correlation between personalized calibration factor, and patient weight, i.e., scattering volume, has been found (*R*^2^ = 0.04). In some case, the patients’ arms are not in the same position during the WBS (arms along the body) and the SPECT/CT (arms above the head) acquisition because of patient comfort issue. Therefore, the 2D attenuation map from the CT does not perfectly match the WBS image leading to a slight underestimation of the activity in the arms. It seems that there is a better agreement between *S*_FAF_ and *S*_std_ when the patients’ arms remain in the same position, despite little activity there.

Repeated acquisition for the standard calibration protocol would have been necessary to evaluate *S*_std_ uncertainty and the relevance of the relative difference between both methods.

## Discussion

The method assumes that all injected activities are present in the planar images. Indeed, image acquisitions must be performed before any biological elimination (in particular urination). Moreover, the method assumes that the activity in the SPECT FOV may be estimated from the planar images. Indeed, WBS and SPECT/CT acquisitions are performed successively within 10 min. We thus assume that the activity redistribution within the body between the two images is negligible. Also, WBS are not corrected from nuclear decay during the acquisitions since they are much faster than the radionuclide half-lives.

Note that the calibration factors reported here are relatively higher than the typical values found in SPECT camera specifications. We indeed observed that enabling the resolution recovery option of the reconstruction algorithm in Xeleris 3.0 leads to larger values. A calibration factor may be involved, but it is not described in the constructor documentation. For patient data, *S*_FAF_ was computed with the average *A*_mean_ over the two-step acquisition rather than one for each step. Since the *A*_mean_ decreases very slightly (less than 0.3%), the error propagated to *S*_FAF_ is negligible. Scatter correction relies on a DEW method but could be applied with other approaches [14].

Dead-time correction with aDT method was important, reducing coefficient variation from 2.4% with sDT to less than 1% with aDT. This may be particulary important for example for ^177^Lu therapies where a large activity (2.5–7.5 GBq) is injected. Here, *ω*_f_ and aDT were determined from ^99m^Tc and applied to ^111^In. Given the injected activities and the low count rate observed in the patient study, the impact of the dead-time correction factors here was negligible (around 1%). A slight dependence of the calibration factor with ^99m^Tc according to the count rate was still observed (< 1%); it may be due to a purely paralyzable model consideration, as recommended in the literature [20], and not a hybrid model.

## Conclusions

We showed that a reliable estimation of the Fraction of Activity in the Field of View, with less than 2% error, may be obtained with the proposed method involving standard planar WBS acquisitions.

Overall relative quantification errors were below 7% in various acquisition conditions. This represents a level of accuracy typically found in the literature for SPECT and PET quantification [1, 6].

The image-based FAF calibration method does not require specific phantom acquisitions and is intrinsically adapted to each acquisition conditions: reconstruction parameters, radionuclides, and attenuation configurations. It could limit systematic bias that could potentially occur with standard calibration protocol. In practice, we advocate the use of those two independent calibration methods, phantom-based and image-based, as quality assurance.
